# Management of donations of personal protective equipment in response to the massive shortage during the COVID-19 health crisis: providing quality equipment to health care workers

**DOI:** 10.1186/s13756-021-01028-0

**Published:** 2021-11-08

**Authors:** Guillaume Cambien, Jérémy Guihenneuc, Xavier Fouassin, Olivier Castel, Anne Bousseau, Sarah Ayraud-Thevenot

**Affiliations:** 1 Hygiene Department, University Hospital of Poitiers, 86021 Poitiers, France; 2 Public Health Department, University Hospital of Poitiers, 86000 Poitiers, France; 3 HEDEX Research Group, INSERM, University Hospital of Poitiers, University of Poitiers, CIC1402, 86021 Poitiers CEDEX, France

**Keywords:** COVID-19, Personal protective equipment, Healthcare workers, Infection control, Crisis management, Healthcare quality improvement, Safety culture

## Abstract

**Background:**

In the COVID-19 pandemic context, a massive shortage of personal protective equipment occurred. To increase the available stocks, several countries appealed for donations from individuals or industries. While national and international standards to evaluate personal protective equipment exist, none of the previous research studied how to evaluate personal protective equipment coming from donations to healthcare establishments. Our aim was to evaluate the quality and possible use of the personal protective equipment donations delivered to our health care establishment in order to avoid a shortage and to protect health care workers throughout the COVID-19 crisis.

**Methods:**

Our intervention focused on evaluation of the quality of donations for medical use through creation of a set of assessment criteria and analysis of the economic impact of these donations.

**Results:**

Between 20th March 2020 and 11th May 2020, we received 239 donations including respirators, gloves, coveralls, face masks, gowns, hats, overshoes, alcohol-based hand rubs, face shields, goggles and aprons. A total of 448,666 (86.3%) products out of the 519,618 initially received were validated and distributed in health care units, equivalent to 126 (52.7%) donations out of the 239 received. The budgetary value of the validated donations was 32,872 euros according to the pre COVID-19 prices and 122,178 euros according to the current COVID-19 prices, representing an increase of 371.7%.

**Conclusions:**

By ensuring a constant influx of personal protective equipment and proper stock management, shortages were avoided. Procurement and distribution of controlled and validated personal protective equipment is the key to providing quality care while guaranteeing health care worker safety.

## Background

The coronavirus disease named COVID-19 (previously known as “2019 novel coronavirus”) by the World Health Organization on 11 February 2020 is caused by a severe acute respiratory syndrome coronavirus 2 (SARS-CoV-2) [1]. The first cases were reported on 31th December 2019 in Wuhan City, Hubei Province of China [2]. SARS-CoV-2 belongs to the family of Coronaviridae, related to enveloped non-segmented positive sense RNA viruses [3]. As of 31th May 2020, the virus had spread to at least 188 countries, about 6,000,000 confirmed cases and 367,000 deaths had been recorded, and since the beginning of April daily cases ranging from 66,000 to 137,000 were identified [4, 5]. Various clinical symptoms have been reported: fever, cough, myalgia or fatigue, expectoration, dyspnoea, headache or dizziness, diarrhoea, nausea and vomiting [6, 7]. Transmission of COVID-19 may occur through direct contact with infected people or through indirect contact with surfaces or objects via exposure of mucosae (mouth and nose) or conjunctiva (eyes) to viral particles. The virus is primarily transmitted between people through respiratory droplet transmission (> 5–10 µm in diameter) and contact routes [8–10]. Airborne transmission (≤ five µm in diameter) has likewise been reported and should be taken into account, especially in specific circumstances such as when aerosol-generating medical procedures (AGMPs) are carried out [11, 12]. As with other communicable diseases, particularly respiratory viruses, the provision and proper use of personal protective equipment (PPE) is one of the main barrier measures protecting health care workers [13]. Indeed, the World Health Organization (WHO) recommends that health care workers wear gowns, gloves, goggles and face masks when no AGMP are in progress or gowns (± aprons if gowns are not fluid-resistant), gloves, goggles and respirators when AGMPs are in progress [14–16].

In this COVID-19 pandemic context, a massive shortage of PPE occurred [17, 18]. Indeed, the appearance of many simultaneous cases of COVID-19 in different countries led to increased activity in health care institutions and to increased consumption of PPE. In addition, widespread quarantines, especially in Asian countries such as China (the main PPE producers) led to a reduction or even a shutdown of production of the supply chains producing these PPEs and to disruption of deliveries [19, 20]. To increase the available stocks of PPE, several countries such as France have appealed for donations to acquire the needed supply from individuals or industries [17, 21].

The University Hospital of Poitiers received many donations in this way. However, these donated materials were often different from the ones commonly used in our health care establishment. None of the previous research has studied how to evaluate personal protective equipment coming from donations to healthcare establishments. We consequently needed to evaluate the quality and the possible use of these donations in health care units, our objectives being to avoid a shortage and to care for patients while keeping our health care workers safe.

## Methods

The donation study period at the University Hospital of Poitiers lasted from 20th March 2020 (reception of the first donation) to 11th May 2020 (end of lockdown in France). An entire floor of secure storage space with heating, venting and air conditioning was made available to receive and store donations according to good storage practice (GSP). Each donation was received and individualized on an identified pallet by the infection control team and storekeepers in order to map the donations and improve storage quality and safety. We applied the World Health Organization’s GSP for medical products and adapted them to donations received [22]. For each donation, several elements were listed: type of product, reference number, batch identification, name of the product, packaging status, number of products, name of the donor, donation receipt date and expiration date. After which, our intervention focused on evaluation of the donations by residents on the infection control team, the objective being to determine whether or not they could be used in our health care units. For all products received, integrity was initially checked. Depending on the products received, the assessment criteria were different. They are detailed in Table 1.

**Table 1:** Assessment criteria for donations of personal protective equipment and other products

Type of personal protective equipment		Assessment criteria
**Head protections**		
	Hats	Covering completely the hair and the ears
	Face Shields	Directly protecting the face
		Stiffness
		Waterproofness
		Headbands (at least 10 mm in width)
		Ease of disinfection
		Product standards
	Goggles	Completely covering the eyes
		Waterproofness
		Ease of disinfection
		Product standards
**Respiratory protections**		
	Face masks	Completely covering the mouth, nose and chin
		Bacterial Filtration Efficiency
		Elastic resistance
		Nose bridge strength
		Product standards
	Respirators (FFP2 or eq. N95 / Korea 1st Class / KN95 / DS2 / P2 / PFF2)	Completely covering the mouth, nose and chin
		Elastic resistance
		Nose bridge strength
		Fit check
		Expiration date
		Uncovered outer valve
		Product standards
**Body and hand protections**		
	Gowns	Completely covering the chest, the arms, the forearms and the legs up to the knees
		Stitching locations
		Waterproofness
		Water repellency
		Product standards
	Coveralls	Completely covering the head (except the face), the chest, the arms, the forearms and the legs up to the feet’s
		Water repellency
		Waterproofness
		Product standards
	Aprons	Completely covering the chest and the thigh
		Waterproofness
	Gloves	Product composition
		Total length
		Resistance
		Expiration date
		Product standards
	Overshoes	Completely covering the shoes
**Others**		
	Alcohol-based hand rubs	Product composition
		Expiration date

For face masks and respirators, we first verified the presence of the CE marking or observance of international standards equivalent to the European AFNOR (Association Française de NORmalisation / French Standardization Association) standard EN 14,683 for face masks and EN 149 for respirators [23, 24]. For face masks, international standards equivalent to the French standard could be observed (American Society for Testing and Materials (ASTM) F2100-19 level 1, 2 or 3; USA / YY/T 0969–2013 or YY 0469–2011; China). For respirators, international standards equivalent to the French standard could be observed (N95 models; USA; NIOSH 42C-FR84 / Korea 1st Class models; Korea; KMOEL -2017–64 / KN95 models; China; GB2626-2006 or 2019 / DS2 models; Japan; JMHLW-Notification 214,2018 / P2 models; Australia; AS/NZS 1716:2012 / PFF2 models; Brazil; ABNT-NBR 13,698:2011 / N95 models; Mexico; NOM-116–2009) [25, 26]. In the absence of CE marking and international standard equivalent, a manufacturer's declaration of conformity had to be provided with: name and address of the manufacturer, product reference, the equivalent international standard and a test report from a laboratory recognized by the nation having issued the standard [25, 27].

If a CE marking, an equivalent international standard or a declaration of conformity was available and if they met the requirements detailed in Table 1, the face masks and respirators were deemed usable; if not, they were returned to the donor. In France, type II face masks with bacterial filtration efficiency against three micron particles equal to or greater than 98% are reserved for health care workers caring for patients with COVID-19 and those with an efficiency of more than 95% (type I) are reserved for patients and others, in view of reducing the risk of spreading infections, particularly in the context of an epidemic or pandemic such as COVID-19 [28].

Upon receipt of expired respirators, we followed the French government recommendations on assessment of their possible use [29]. Several points had to be checked, such as integrity of the packaging and appearance (original colour) of the mask by means of visual inspection, assessment of strength of the elastics and nasal support bar used, and a fit check test. Due to the lack of specific recommendations, we chose to test two percent of each received batch, as when controlling solid sterile pharmaceutical substances [30].

For coveralls and gowns, we first checked for the CE marking, the European standard and the marking via the presence of a specific pictogram [31]. When there was no applicable standard, we were unable to carry out the tests recommended by AFNOR or ASTM international (American Society for Testing and Materials) [31, 32]. According to the relevant guidelines, a viral penetration test with hydrostatic pressure from 3.5 kPa to 345 kPa is required. Hence, on both counts, we decided to evaluate water repellency by flowing a continuous stream of water on the tested samples and to assess waterproofness by exerting pressure with water corresponding to clinical use. If it failed both tests, the product was deemed unusable. The water-repellent gowns were used for entrance in the patient environment and the waterproof gowns were used when contact and close care of the patient was required. Coveralls were used mainly for the staff mobilized in COVID-19 drive tests and in the emergency department.

To select gloves, we first checked for the standard and the marking via the presence of a specific pictogram [33]. As recommended by the Food and Drug Administration, we excluded non-medical (or non-standard) gloves and those with powder [34]. Glove length was also measured and a cut-off point was set between gloves shorter than 300 mm (gloves for standard precautions) or longer than 300 mm (gloves for the COVID-19 care unit). Glove composition was also recorded.

The measures to evaluate our intervention were stock levels of products received and percentage of products usable in the institution. We also evaluated percentages of products not usable and returned to the donor and percentages of products not usable and destroyed due to delivery of defective or degraded products.

The economic aspect of the donations was evaluated by calculating the total budget of all donations and price evolution based on the prices we paid in our central purchasing department in January 2020 and the price in July 2020.

Quantitative variables were expressed as means ± standard deviation (SD); qualitative variables were presented as frequencies and percentages. Qualitative variables were compared using chi-square test. Analyses were performed with R statistical software (R Core Team, 2019) [35]. A two-sided alpha-level of 0.05 was chosen for statistical significance of all analyses.

This article was written according to the SQUIRE reporting guidelines designed to improve the quality, value and safety of healthcare [36].

## Results

**Fig. 1:**
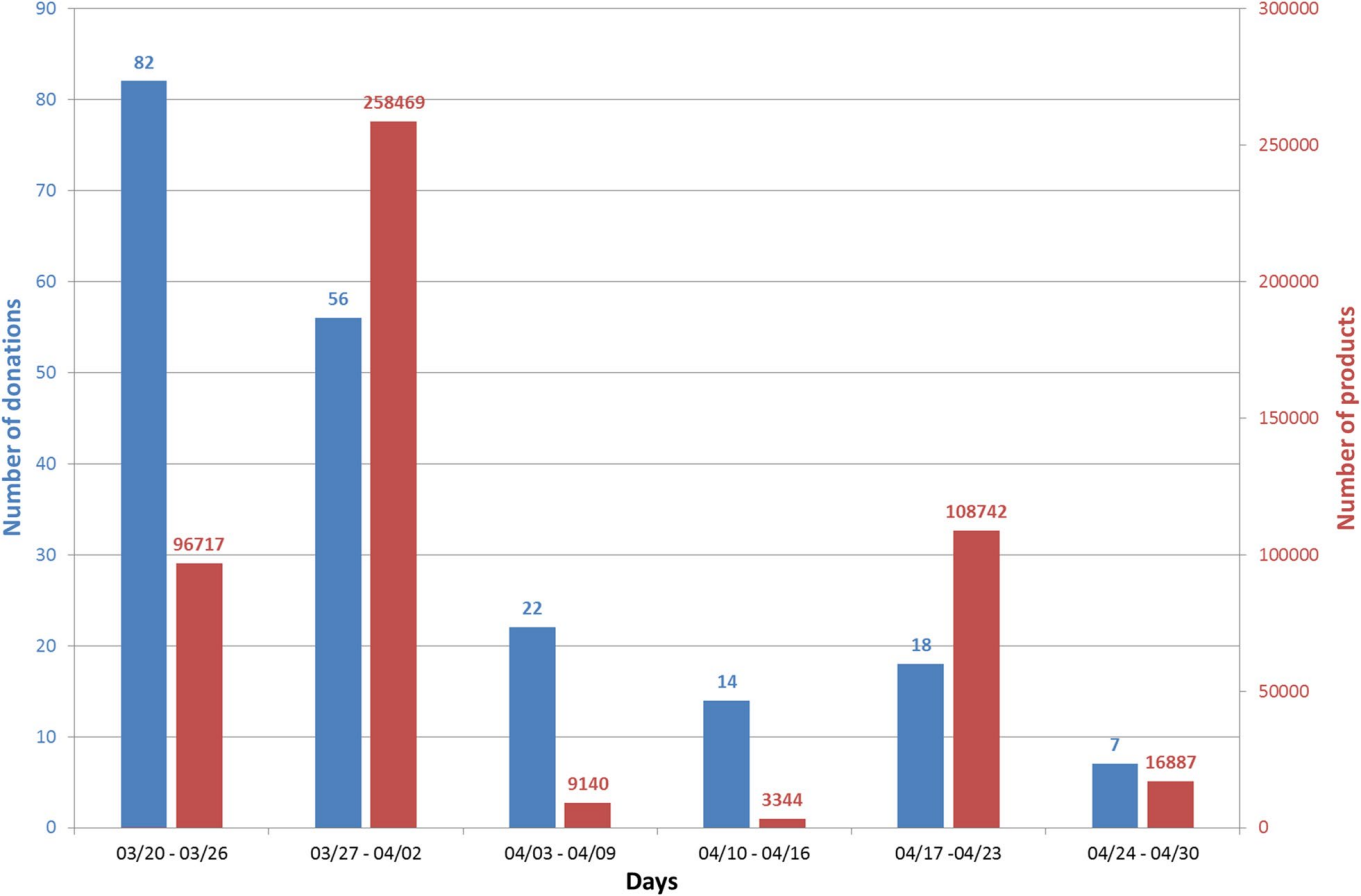
Evolution of donations collected at the University Hospital of Poitiers. Donations collected from 20th March 2020 to 11th May 2020; (missing data n = 40 donations (16.7%); data expressed as n

During the study period (from 20th March 2020 to 11th May 2020), we received a total of 239 donations corresponding to a total number of 519,618 products (one donation corresponds to a given type of product). The number of products per donation was equal to 2174 ± 12,476 products on average. The number of donations and products donated throughout the study period is reported in Fig. 1.

The number of donations was highest during the first week with 82 donations received and decreased over time to seven donations at the end of April. During the periods from 27th March to 02nd April and from 17th April to 23th April, the two largest donations were received, with 160,000 and 100,000 face masks, respectively. No donation was received from 1st May to 11th May (data not shown).

**Fig. 2:**
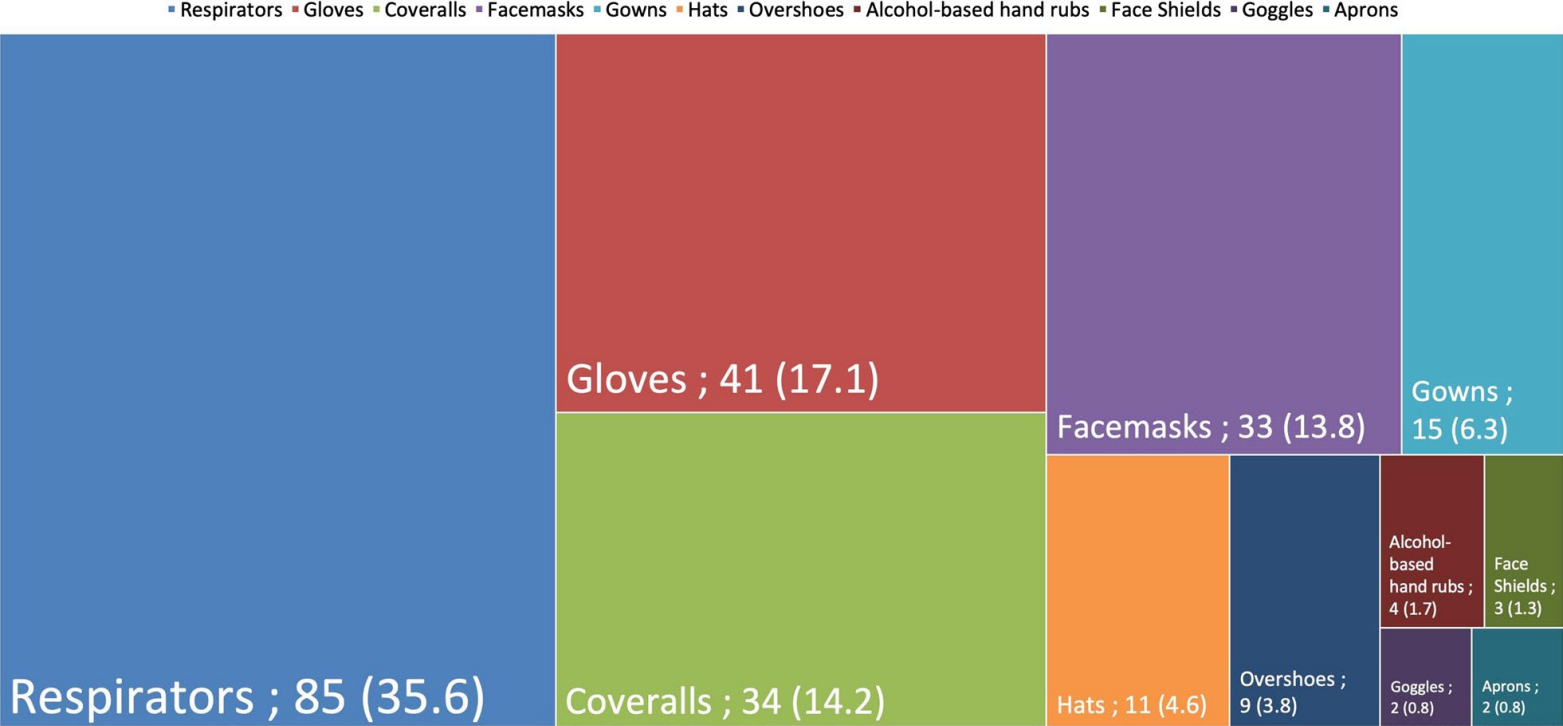
Distribution of the types of donations received. Data expressed as n and percentages

Several types of donations were received during this period. The results are indicated in Fig. 2.

The five main types of donations were respirators, gloves, coveralls, face masks, and gowns with 85 (35.6%), 41 (17.1%), 34 (14.2%), 33 (13.8%) and 15 (6.3%), respectively. However, considering the number of products given, the five types of products most frequently received were face masks, respirators, hats, gloves and overshoes with 309,110, 122,296, 43,989, 30,688, and 6700, respectively. On average we received 9367 ± 32,150 face masks, 1439 ± 3631 respirators, 3999 ± 8099 hats, 748 ± 1063 gloves, 744 ± 1063 overshoes, 68 ± 114 coveralls and 105 ± 91 gowns per donation.

**Fig. 3:**
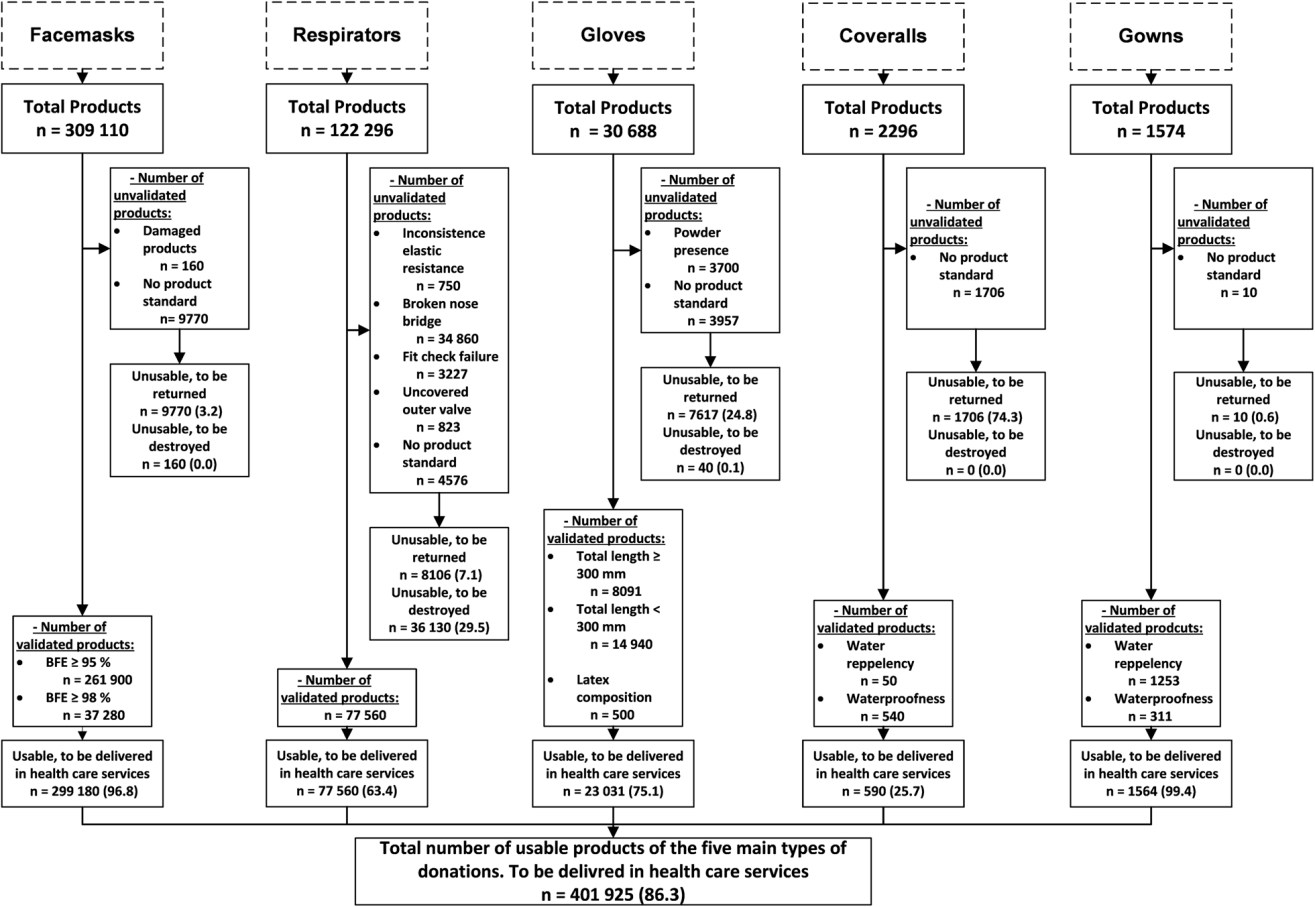
Evaluation of the five main types of donations received. Data expressed as n and percentages

Finally, we evaluated all donations, and the results for the five main types of donations are presented in Fig. 3.

Following evaluation of the donations received, a total of 448,666 (86.3%) out of the 519,618 products initially received were distributed in health care units eq. to 126 out of a total of 239 donations (52.7%), 34,622 products were deemed unusable in our healthcare establishment and were returned to donors (6.7%) eq. to 98 donations (41.0%) and 36,330 were of poor quality or damaged, and were destroyed (7.0%) eq. to 15 donations (6.3%). Small donations composed of less than 200 products were validated less frequently (42 out of 119 donations validated (35.3%)) than larger donations (equal to or more than 200 products) with 84 donations out of 120 donations validated (70.0%) (p < 0.001).

Regarding respirators, the validation process was difficult. Indeed, 69 donations (81.2%) eq. to 121,493 products (99.3%) received have passed their expiration date. It was therefore necessary to check them according to the points stated above. Broken nose bridge, absence of product standard and fit check failure were the most frequent reasons for invalidation.

Regarding other donations, we validated all aprons and face shields with 600 and 1190 products received, respectively. Alcohol-based hand rubs and hats were also frequently validated, with 1142 out of a total of 1146 (99.7%) and 43,789 out of a total of 43,989 (99.6%), respectively. We received only 29 goggles and validated 20 of them (69.0%). Finally, we did not use overshoes in our COVID-19 care unit, and since these products were too brittle to be used in other care units, we decided to return the 6700 products received to the donors.

Finally, we evaluated the economic aspect resulting from donations, and the results are presented in Table 2.

**Table 2:** Economic aspect of the validated donations

Products	Number of validated products	January 2020		July 2020		Price evolution per unit	Price evolution (%)
		Unit price	Budget of donations	Unit price	Budget of donations		
Respirators	77,560	0.128	9928	1.260	97,726	+ 1.132	884%
Gloves for COVID-19 care unit	8091	0.144	1165	0.257	2079	+ 0.113	78%
Gloves for standard precautions	14 940	0.042	627	0.046	687	+ 0.004	10%
Coveralls	590	7.266	4287	7.770	4584	+ 0.504	7%
Face masks type I	261,900	0.035	9167	0.035	9167	+ 0.000	0%
Face masks type II	37,280	0.034	1268	0.070	2610	+ 0.036	106%
Water-repellent gowns	1253	0.183	229	0.596	747	+ 0.413	226%
Waterproof gowns	311	0.370	115	2.960	921	+ 2.590	700%
Hats	43,789	0.012	525	0.014	613	+ 0.002	17%
Alcohol-based hand rubs	1142	1.190	1359	1.190	1359	+ 0.000	0%
Face Shields	1190	3.500	4165	1.356	1614	− 2.144	− 61%
Goggles	20	1.120	22	1.140	23	+ 0.020	2%
Aprons	600	0.025	15	0.080	48	+ 0.055	220%
Total	448,666	–	32,872	–	122,178	–	–

Price expressed as euros

The total donation budget was 32,872 euros when applying pre COVID-19 prices and 122,178 euros when applying the current COVID-19 prices (a 371.7% increase). Mean price evolution was 168%. Indeed, the unit prices of respirators and waterproof gowns were multiplied by a factor of almost 10, soaring from 0.128 to 1.260 euros, and by a factor of 8, from 0.370 to 2.960 euros, respectively. According to WHO recommendations, the cost of PPE to care for COVID-19 patients without AGMPs, (face mask type II + water repellent gown + goggles + gloves for COVID-19 care unit) increased from 1.481 in January to 2.063 euros in July 2020; with AGMPs (respirator + water repellent gown + apron + goggles + gloves for COVID-19 care unit), it was multiplied by more than a factor of two, growing from 1.600 in January to 3.333 euros in July 2020.

## Discussion

We report the results on management of the donations received by the University Hospital of Poitiers during the COVID-19 health crisis. For each class of products, we have constructed original assessment criteria based on national and international standards, the objective being to validate or invalidate the use of donations in a health care establishment. Our validation system enabled us to handle a massive influx of donations from different donors, to process and evaluate each donation individually and, finally, to validate 448,666 out of 519,618 total products (86.3%) from 126 out of 239 donations (52.7%) for use in the establishment. Smaller donations were less often validated for institutional use, which explains the difference between number of validated products and number of validated donations (p < 0.001). Regarding respirators, we validated 77,560 (63.4%) out of a total of 122,296 products. This percentage is consistent with another study in which the effectiveness of non-CE-marked respirators was evaluated [37]. Notwithstanding a massive worldwide shortage, our efforts allowed us to avoid any PPE shortage within our establishment. Assessment of the different products prevented the distribution of unsuitable products to our health care workers, who could consequently provide quality care while their safety was guaranteed.

In addition, the generosity of donors was highlighted through the economic study. According to market prices, donations currently represent a total budget of 122,178 euros. Variations of PPE market prices were studied by our central purchasing office with a mean price increase of 168% from before the crisis until today. This remains pronouncedly lower than in the United States, where the Society for Healthcare Organization Procurement Professionals noted a mean price evolution exceeding 1000% for equivalent products [38].

Throughout this work, limits have been identified. Management of donations was a lengthy and difficult process, involving requisition for one and a half months of two full-time residents from the infection control team. In fact, due to a lack of time explained by the rapid onset of an unprecedented health crisis, donations were not properly planned and coordinated and therefore required multiple controls [39]. For each donations received, the infection control team evaluated PPE performance (see Table 1). The tests recommended in accordance with some of the standards were occasionally too complicated to be carried out at the University Hospital of Poitiers, and at times it was necessary to carry out “homemade tests” to validate the use of some donations. In the case of gowns, for example, we carried out tests simulating working conditions as close as possible to those existing in the care units, thereby guaranteeing products adapted to care. In our study, we did not use indicators to assess the PPE performance in the health care unit. However, to date we have not received any return of validated PPE from the health care unit, or any declaration regarding PPE not adapted to patient care. Regarding nosocomial transmission, it is difficult to establish a link with the wearing of donated PPE. However, the evaluated PPE was mostly distributed in our COVID-19 care units, while most nosocomial COVID-19 infections occurred in other units.

With this work, we have completed a process aimed at overcoming a massive shortage of PPE. However, this approach alone cannot guarantee constant PPE influx and correct stock management. It is therefore necessary to preserve PPE by reducing its use to what is strictly necessary according to international recommendations and by limiting the number of situations potentially exposing caregivers [40]. With this objective in mind, methods for equipment reuse have emerged during the crisis and been shared on a website [41]. In our establishment, we initially reused single-use waterproof gowns after washing them and in the literature, we have observed methods for the treatment of respirators [42–44]. Finally, the Centers for Disease Control and Prevention (CDC) have created a PPE Burn Rate Calculator composed of a spreadsheet-based model that will help health care facilities to plan and optimize PPE use [45]. To protect health care workers, communication of information is essential to enable all concerned parties to know, understand and adapt their care practices in order to ensure PPE compliance with infection prevention control [46–48]. By following all of these recommendations, it is possible, as shown in several studies [49, 50], to provide care to COVID-19 patients without getting infected.

In the context of this work and the health crisis, the complexity and heterogeneity of the different national standards for PPE used in the same indications has rendered the management and processing of donations from one country to another increasingly complicated [51]. In France, before this crisis it was impossible to use respirators other than FFP2 models (commonly used in Europe) [24]. Due to the massive shortage, the French government authorized for a limited period of time the use of respirators with different standards, which could come from seven different countries/regions of the world with very similar microorganism filtration performances [25]. However, studies have shown that there will be more and more health crises in the future, and an increase in PPE donations between countries is to be expected [52]. As a result, we can question the interest of different PPE standards to protect against the same bacteriological or viral risks. It appears important to uniformize standards, to achieve international labelling, and consequently to use equipment uniformly validated by all countries for the sake of the health of one and all.

## Conclusion

By ensuring a constant influx of personal protective equipment and proper management of stocks, shortages in University Hospital of Poitiers did not occur. Procurement and distribution of controlled and validated personal protective equipment is the key to providing quality care while guaranteeing health care worker safety.

## Data Availability

All data generated or analysed during this study are included in this published article.

## Abbreviations

AFNOR: Association Française de NORmalisation/French Standardization Association
AGMPs: Aerosol-generating medical procedures
ASTM: American Society for Testing and Materials
CDC: Centers for Disease Control and Prevention
COVID-19: Coronavirus disease
GSP: Good storage practice
PPE: Personal protective equipment
SARS-CoV-2: Severe acute respiratory syndrome coronavirus 2
WHO: World Health Organization
